# Supplementary material for: Zearalenone Promotes Uterine Hypertrophy through AMPK/mTOR Mediated Autophagy
Source: Toxins (Basel). 2024 Feb 1;16(2):73. doi: 10.3390/toxins16020073 (PMC10892946; doi:10.3390/toxins16020073)

Zearalenone Promotes Uterine Hypertrophy through AMPK/mTOR Mediated Autophagy

Lijie Yang, Wenshuang Liao, Jiuyuan Dong, Xiangjin Chen, Libo Huang, Weiren Yang and Shuzhen Jiang

Table S1. Ingredients and nutrient contents of the basal diet (air-dry basis), %.

Ingredients	Content	Nutrients	Analyzed values
Expanded corn	64.43	Digestible energy, MJ/kg	14.53
Whey powder, CP 3%	5.00	Metabolizable energy，MJ/kg	13.86
Fermented soybean meal	14.00	Crude protein	18.48
Expanded soybean	8.50	Calcium	0.74
Fish meal, CP 63.28%	4.00	Total phosphorus	0.62
$CaHPO_4$	1.15	STTD phosphorus	0.41
Pulverized Limestone	0.70	ATTD phosphorus	0.38
NaCl	0.20	Lysine	1.38
L-Lysine HCl	0.76	Methionine	0.40
DL-Methionine	0.08	Sulfur amino acid	0.66
L-Threonine	0.16	Threonine	0.85
L-Tryptophan	0.02	Tryptophan	0.23
Premix[1)]	1.00		
Total	100.00		

Notes: [1)]Supplied per kilogram of diet: vitamin A, 3300 IU; vitamin D_3, 330 IU; vitamin E, 24 IU; vitamin K_3, 0.75 mg; vitamin B_1, 1.50 mg; vitamin B_2, 5.25 mg; vitamin B_6, 2.25mg; vitamin B_{12}, 0.026 mg; pantothenic acid, 15.00 mg; niacin, 22.50 mg; biotin, 0.075 mg; folic acid, 0.45 mg; Mn ($MnSO_4 \cdot H_2O$), 4.00 mg; Fe ($FeSO_4 \cdot H_2O$), 90 mg; Zn ($ZnSO_4 \cdot H_2O$), 90 mg; Cu ($CuSO_4 \cdot 5H_2O$), 6.00 mg; I (KIO_3), 0.14 mg; Se (Na_2SeO_3), 0.30 mg.

Table S2 The condition of RT reaction.

Ingredients	Volume
5 × Evo M-MLV RT Master Mix	4 μL
Total RNA	<1000 ng/RNA
RNase Free water	up to 20 μL (16-1000/RNA)

Table S3 Primers sequences of qRT-PCR.

Target genes	Primer sequence（5’-3’）	Product size bp	Location
β-actin	F: GGACTTCGAGCAGGAGATGG<br>R: AGGAAGGAGGGCTGGAAGAG	138	XM_021086047.1
BAX	F: AGGCGGGGTTTCATCCA<br>R: AGACACTCGCTCAACTTCTTGGTAG	110	XM_013998624.2
BCL-2	F: CCTACCACCACCACCTCTTAC<br>R: TGAGTCAGTCTAGCCAACAACAT	90	XM_021099593.1
PCNA	F: GAGGAGGAAGCAGTTACCATAGA<br>R: GACATACTGAGTGTGACTGTAGGA	119	NM_001291925.1
ATG5	F: CCGCAACCAACAGATTGAAGGA<br>R: CCTCCACCAAACCTGACTGAAG	107	NM_001037152.2
ATG7	F: GACCATGCCATGATTGGACCTGAG<br>R: GCCTGTCAACCTTCTCGCTGTC	133	NM_001190285.1
ATG9	F: CCTCGCTCACATCCACTACAT<br>R: GGCTCAGTAACTCCTCCAAGAT	125	NM_001190275.1
Beclin1	F: GGTGTCTCTCGCAGATTCATCC<br>R: TCTTCGGCTGAGGTTCTCCAT	117	NM_001037152.2
LC3	F: GCCTTCTTCCTGCTGGTGAAC<br>R: ATCCTCATCCTTCTCCTGCTCAT	87	NM_001170827.1

Notes: BAX, BCL2 associated X; BCL-2 , B-cell lymphoma-2; PCNA, Proliferating cell nuclear antigen; AMPK, AMP-activated protein kinase; mTOR, mammalian target of rapamycin; ATG5, Autophagy related 5; ATG 7, Autophagy related 7; ATG 9, Autophagy related 9; LC3, Microtubule associated protein 1 light chain 3 alpha.

Table S4 The condition of qRT-PCR.

Ingredients	Volume
2X SYBR Green Pro Taq HS Premix	10 μL
Forward primer (10 μM)	0.4 μL
Reverse primer (10 μM)	0.4 μL
cDNA	2 μL
RNase free water	7.2 μL

Table S5 Correlation between uterine toxins content and the relative protein expression of autophagy and proliferation related genes.

Items	ZEA, ng/g	α-zearalenol, ng/g	β-zearalenol, ng/g
p-AMPK/AMPK	0.909**	0.907**	0.907**
p-mTOR/mTOR	-0.919**	-0.920**	-0.920**
Beclin1	0.956**	0.955**	0.955**
LC3II/I	0.916**	0.918**	0.918**
ATG5	0.886**	0.881**	0.881**
ATG7	0.967**	0.965**	0.965**
ATG9	0.971**	0.968**	0.968**
PCNA	0.986**	0.987**	0.987**
BCL2	0.900**	0.896**	0.896**
BAX	-0.951**	-0.952**	-0.952**

Notes: BAX, BCL2 associated X; BCL-2 , B-cell lymphoma-2; PCNA, Proliferating cell nuclear antigen; AMPK, AMP-activated protein kinase; mTOR, mammalian target of rapamycin; ATG5, Autophagy related 5; ATG 7, Autophagy related 7; ATG 9, Autophagy related 9; LC3, Microtubule associated protein 1 light chain 3 alpha.

Table S6. Correlation of the relative protein expression among the relative protein expression of AMPK/mTOR, proliferation and autophagy related genes.

**Items.**	**p-AMPK /AMPK**	**p-mTOR /mTOR**	**Beclin1**	**LC3II/I**	**ATG5**	**ATG7**	**ATG9**	**PCNA**	**BCL2**
p-mTOR/mTOR	-0.918**								
Beclin1	0.943**	-0.944**							
LC3II/I	0.786**	-0.893**	0.879**						
ATG5	0.884**	-0.775**	0.876**	0.726**					
ATG7	0.903**	-0.876**	0.917**	0.811**	0.924**				
ATG9	0.944**	-0.916**	0.957**	0.869**	0.952**	0.961**			
PCNA	0.875**	-0.877**	0.933**	0.861**	0.887**	0.966**	0.952**		
BCL2	0.929**	-0.821**	0.920**	0.741**	0.975**	0.934**	0.953**	0.929**	
BAX	-0.907**	0.948**	-0.930**	-0.953**	-0.800**	-0.880**	-0.923**	-0.898**	-0.834**

Notes: BAX, BCL2 associated X; BCL-2 , B-cell lymphoma-2; PCNA, Proliferating cell nuclear antigen; AMPK, AMP-activated protein kinase; mTOR, mammalian target of rapamycin; ATG5, Autophagy related 5; ATG 7, Autophagy related 7; ATG 9, Autophagy related 9; LC3, Microtubule associated protein 1 light chain 3 alpha.

.

Table S7. Effect of zearalenone on the relative mRNA expression of p-AMPK/AMPK, p-mTOR/mTOR, autophagy and proliferation apoptosis related genes in the porcine endometrial epithelial cells.

Items	Treatment				*P* value	
	Control	ZEA5	ZEA20	ZEA40	Treatment	Linear
ULK1	7.23 ± 0.10[c]	7.34 ± 0.14[c]	8.46 ± 0.07[b]	10.06 ± 0.21[a]	<0.001	<0.001
TSC1	10.17 ± 0.28	10.01 ± 0.03	9.85 ± 0.14	9.96 ± 0.19	0.673	0.626
TSC2	6.06 ± 0.17[c]	6.15 ± 0.12[c]	7.66 ± 0.08[b]	9.36 ± 0.01[a]	<0.001	<0.001
Rheb	181.94 ± 0.51[a]	180.27 ± 0.21[a]	162.59 ± 1.44[b]	151.27 ± 0.46 [c]	<0.001	<0.001
Beclin1	35.67 ± 0.37[c]	35.96 ± 0.18[c]	41.32 ± 0.06[b]	45.17 ± 0.17[a]	<0.001	<0.001
LC3	59.13 ± 1.68[c]	61.03 ± 1.88[c]	79.73 ± 1.80[b]	154.01 ± 1.21[a]	<0.001	<0.001
ATG5	6.31 ± 0.08[c]	6.34 ± 0.04[c]	7.37 ± 0.01[b]	8.27 ± 0.07[a]	<0.001	<0.001
ATG7	1.12 ± 0.07[c]	1.14 ± 0.01[c]	1.69 ± 0.01[b]	1.90 ± 0.04[a]	<0.001	<0.001
ATG9	0.20 ± 0.01[c]	0.22 ± 0.02[c]	0.45 ± 0.01[b]	0.61 ± 0.03[a]	<0.001	<0.001
PCNA	190.80 ± 0.05[b]	195.73 ± 0.20[a]	184.49 ± 0.79[c]	106.53 ± 1.64[d]	<0.001	<0.001
BCL2	0.65 ± 0.01[b]	0.83 ± 0.01[a]	0.60 ± 0.01[c]	0.56 ± 0.01[d]	<0.001	0.008
BAX	82.63 ± 0.77[c]	76.95 ± 0.77[d]	86.54 ± 1.10[b]	90.65 ± 1.21[a]	<0.001	<0.001

Notes: BAX, BCL2 associated X; BCL-2 , B-cell lymphoma-2; PCNA, Proliferating cell nuclear antigen; AMPK, AMP-activated protein kinase; mTOR, mammalian target of rapamycin; ATG5, Autophagy related 5; ATG 7, Autophagy related 7; ATG 9, Autophagy related 9; LC3, Microtubule associated protein 1 light chain 3 alpha; Rheb, Ras homologue enriched in brain; TSC1, tuberous sclerosis protein 1; TSC2, tuberous sclerosis protein 2; ULK1, UNC-51-like kinase 1.

Figure S1 Effects of ZEA on cells viability of porcine endometrial epithelial cells.

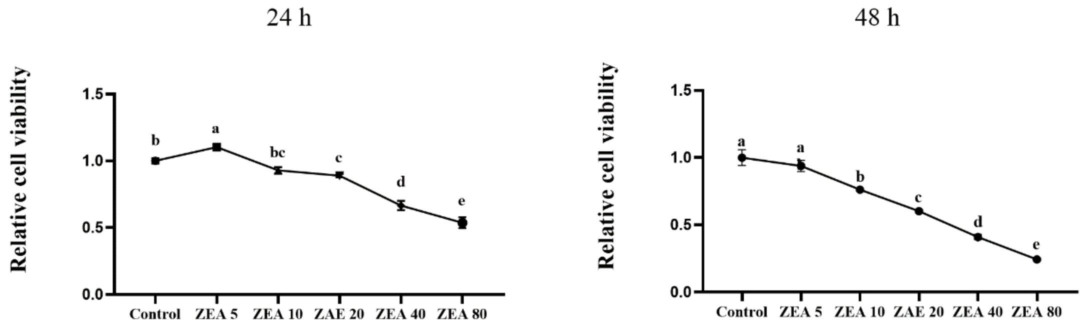

Figure S2 Effects of zearalenone (ZEA) on the protein relative expression of related genes in the uterus of gilts.

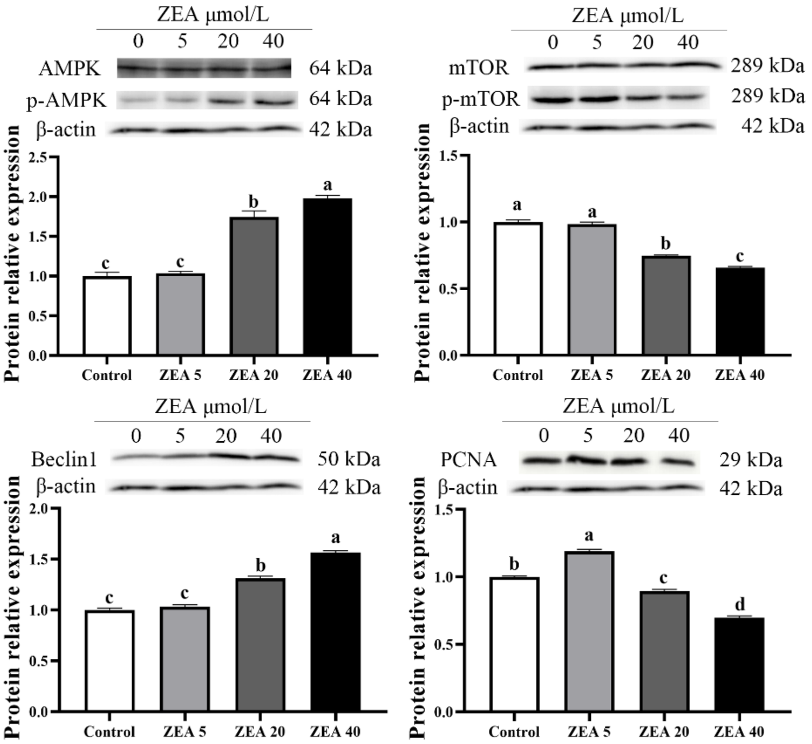

Supplement: Supplementary file 1 [file toxins-16-00073-s001.zip › toxins-2778644-supplementary.pdf]
